# Radiological differentiation between bacterial orbital cellulitis and invasive fungal sino-orbital infections

**DOI:** 10.1007/s10792-024-03241-3

**Published:** 2024-07-08

**Authors:** Terence Ang, Wanyin Lim, Viraj Chaggar, Sandy Patel, Dinesh Selva

**Affiliations:** 1 The University of Adelaide, Adelaide, SA Australia; 2 Department of Ophthalmology, The Royal Adelaide Hospital, Adelaide, SA 5000 Australia; 3 Department of Medical Imaging, The Royal Adelaide Hospital, Adelaide, SA Australia; 4 Jones Radiology, Adelaide, SA Australia

**Keywords:** Fungal infection, MRI, Orbital infection, Orbital cellulitis, Sino-orbital fungal infection

## Abstract

**Purpose:**

Invasive fungal orbital infections (IFOI) may be difficult to differentiate from sinogenic bacterial orbital cellulitis (OC). This study investigates the features differentiating OC from IFOI on magnetic resonance imaging (MRI).

**Methods:**

Retrospective study of adult patients with sinogenic OC and IFOI with pre-intervention MRI. Patients without post-septal involvement, non-sinogenic OC (e.g.: secondary to trauma) and poor-quality scans were excluded. Independent Sample’s *t* test and Fisher’s exact test were conducted with *p* < 0.05 deemed statistically significant.

**Results:**

Eleven cases each of OC (Mean age: 41.6 ± 18.4 years-old, Male: 10) and IFOI (Mean age: 65.0 ± 16.6 years-old, Male: 9) between 2006 and 2023. IFOI patients were older, more likely immunocompromised and had a lower mean white-cell count (*p* value = 0.005, 0.035 and 0.017, respectively). The ethmoid and maxillary sinuses were most commonly involved in both entities. Pre-septal and lacrimal gland involvement were more common in OC (*p* = 0.001 and 0.008, respectively). Infiltrative OC orbital lesions were poorly demarcated, whilst those in IFOI were expansile/mass-like invading the orbit from the adjacent paranasal sinuses. Specific IFOI features included loss-of-contrast-enhancement (LoCE) of paranasal sinus tissues with orbital extension. Extra-orbital and -sinonasal extension indicative of IFOI included contiguous skull base or pterygopalatine fossa involvement, retro-antral and masticator space stranding and vasculitis.

**Conclusion:**

This study describes the key MRI features of IFOI including differentiating markers from OC. These specific features, such as LoCE of the paranasal and orbital soft tissues, the location and pattern of contiguous soft-tissue involvement, provide expedient identification of IFOI which necessitate early surgical intervention for microbiological confirmation of an invasive fungal pathology.

## Introduction

Invasive fungal orbital infections (IFOI) typically originate from the paranasal sinuses, and secondary orbital involvement may result via contiguous spread and is associated with a poor morbidity and mortality [1, 2]. Delayed diagnosis and initiation of selective antifungal therapy can have disastrous implications [1]. Misdiagnosis of IFOI is common as a range of infective and inflammatory conditions may mimic its clinico-radiological presentation [3, 4]. Bacterial orbital cellulitis (OC) may occur secondary to acute sinusitis and clinical presentation may be similar to IFOI. Prior studies have explored differentiation of IFOI and OC on computed tomography (CT) with various limitations, however magnetic resonance imaging (MRI) provides superior delineation of the orbital soft-tissue structures [2, 5–7]. This study aims to investigate and review the features which differentiate OC from IFOI on MRI.

## Methods

### Subjects

This was a retrospective comparative study of adult patients (> 18 years old) diagnosed with sinogenic bacterial OC and invasive fungal orbital infections who underwent a pre-intervention MRI. Patients without evidence of post-septal involvement, non-sinogenic OC (e.g.: secondary to trauma) and poor-quality MRI scans were excluded.

Patients were identified from the Oculoplastic Unit at the Royal Adelaide Hospital (Adelaide, Australia). Data collected included patient demographics, clinical presentation, laboratory investigations (white-cell count, C-reactive protein and relevant microbiological analysis), MRI features, management and clinical outcomes. All research was conducted in accordance with the Declaration of Helsinki and was approved by the Central Adelaide Local Health Network Human Research Ethics Committees (CALHN HREC) with a waiver of consent provided (Reference number: 18536).

### Image acquisition and analysis

MRI scans of the paranasal sinuses, orbits and brain were performed on either 1.5-Tesla or 3.0-Tesla scanners. Sequences conducted included a combination of T1- and T2-weighted imaging (T1 and T2), fat-supressed contrast-enhanced T1 (T1 FS CE), fat-supressed T2 (T2 FS) and diffusion weighted imaging (DWI) with apparent diffusion coefficient (ADC) mapping. Scans were reviewed by two experienced radiologists (SP and WL), noting the absence or presence, and pattern of orbital involvement (e.g., extraocular muscle, lacrimal gland, orbital apex, optic nerve and sheath involvement) and regional (e.g., paranasal sinuses) structures.

### Statistical analysis

Statistical analysis was performed with SPSS (IBM Corporation, New York). Where applicable, results are expressed as means ± standard deviation (σ) and presented in relevant tables. Differences in means were analysed by the Independent Sample’s *t* test with *p* < 0.05 deemed statistically significant. Fisher’s exact test was used to analyse clinical and radiological associations between IFOI and OC.

## Results

### Patient demographics and clinical features

Twenty-two MRI scans from twenty-two patients presenting between 2006 and 2023 were included. Seven IFOI and ten OC cases had contrast-enhanced scans, and eight IFOI and ten OC cases also had DWI. Table 1 summarises the patient demographics.

**Table 1:** Summary of patient demographics and clinical features

	Bacterial orbital cellulitis	Invasive fungal orbital infection	*P *value
Number of patients	11	11	
Mean age ± σ (years)	41.6 ± 18.4	65.0 ± 16.6	0.005
Male: Female	10:1	9:2	1
*Clinical features*			
Mean duration of symptoms (days)	3	23.4	0.063
Immunocompromise	0	5	0.035
Diabetes mellitus	1	4	0.311
Orbital/ocular pain	10	10	1
Periorbital erythema and/or oedema	11	7	0.214
Proptosis	8	7	1
Visual compromise (i.e. reduced VA or optic neuropathy)	4	6	0.670
Systemic symptoms*	3	4	1
*Laboratory*			
Mean white-cell count	17.9 ± 7.3	10.6 ± 5.5	0.017
Mean C-reactive protein	149.8 ± 84.4	91.5 ± 106.3	0.212

*Systemic features included symptoms such as fever, headaches, malaise, and fatigue

All cases of OC and IFOI were treated with appropriate antimicrobial therapy (i.e., antibiotics or antifungals). Of the patients with IFOI, seven patients had an initial suspicion of an invasive fungal pathology due to risk-factors (e.g.: immunocompromise), clinical features (e.g.: necrotic tissue on naso-endoscopy) and extensive radiological features. In the remaining four patients, there was initial diagnostic uncertainty, with diagnoses including pre-septal cellulitis, bacterial orbital cellulitis or a metastatic neoplastic process. A diagnosis of IFOI was subsequently made following clinical deterioration or poor response to isolated antibiotic therapy which prompted repeat imaging and/or surgical intervention. Notably, all IFOI cases received empirical antibiotics at some time during the course of their treatment. Eleven cases (100%) of OC and nine cases (81.8%) of IFOI required surgical intervention. Nine OC cases compared with two IFOI cases proceeded to have complete clinical resolution with no residual ocular symptoms. One OC patient had vision loss to hand movements. Visual prognosis was poor in IFOI, with three patients proceeding to exenteration/enucleation, three patients experiencing visual compromise (i.e., persistent optic neuropathy) and one patient death. One case of OC and IFOI each, were lost to follow-up and long-term outcomes not recorded.

### Radiological features

**Fig. 1:**
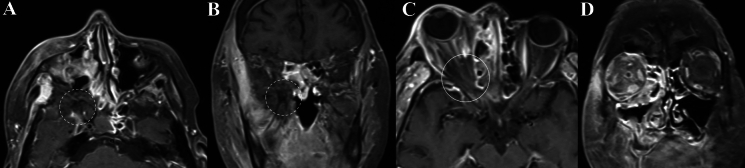
Orbital imaging of a 66-year-old female with invasive mucormycosis involving the right orbit. **A** and **B** Demonstrate non-enhancement of the right sphenoid sinus and orbital apex contiguous with the pterygopalatine fossa and skull base with surrounding tissue contrast-enhancement on axial and coronal T1FS CE (Dashed circle). **C** Demonstrates the LoCE in the EOM of the orbital apex (Solid circle) on axial T1 FS CE with right-sided proptosis. **D** Demonstrates right sphenoid and ethmoid contrast-enhancement, opacification and mucosal thickening, and right-sided EOM enlargement, and diffuse contrast-enhancement in the right orbital fat on coronal T1 FS CE

**Fig. 2:**
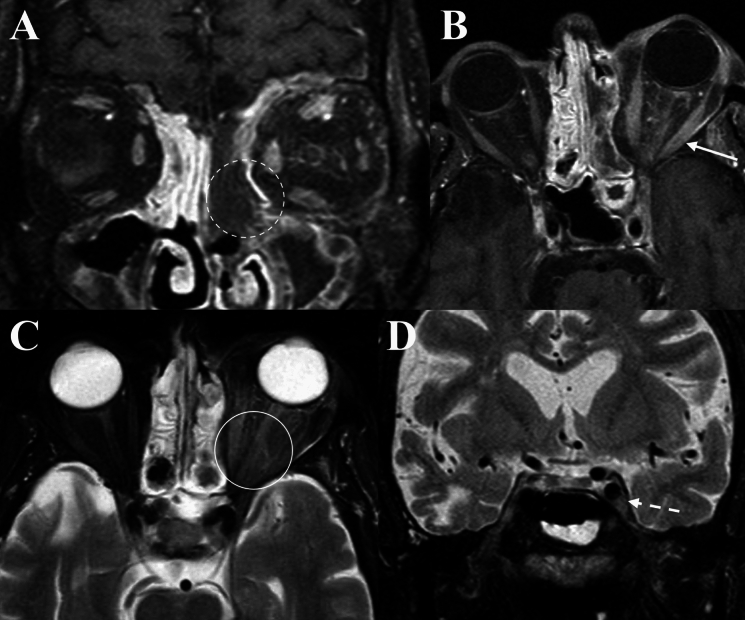
Orbital imaging of a 55-year-old male with invasive mucormycosis involving the left orbit. **A** and **B** Demonstrates LoCE of the left superior and middle turbinates (“Black turbinate” sign), and the ethmoidal mucosa (Dashed circle). There is proptosis and diffuse heterogenous contrast-enhancement of the left orbit, along with mild EOM enlargement and contrast-enhancement (Solid arrow). **C** Also depict the respective changes of the orbital fat and EOM on axial T2 FS (Solid circle). **D** Coronal T2FS images demonstrating asymmetric expansion of the left cavernous sinus (Dashed arrow)

**Fig. 3:**
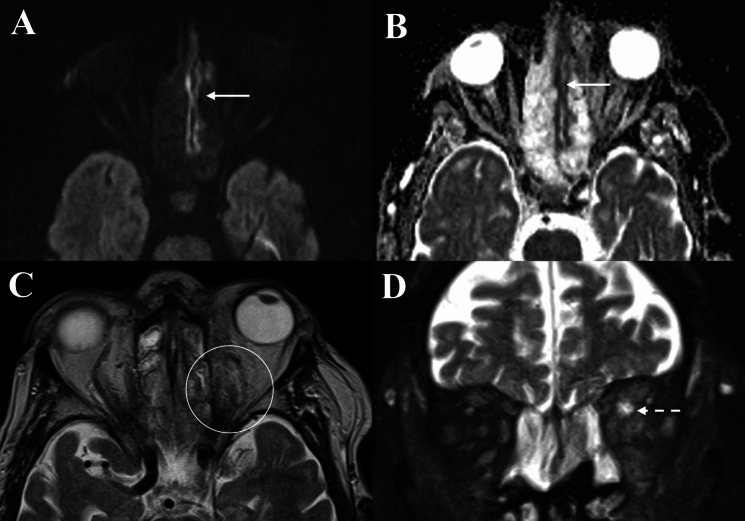
Orbital imaging of a 75-year-old male with invasive mucormycosis involving the left orbit. **A** and **B** Demonstrate regions of diffusion restriction along the mucosa lining of the ethmoid sinus on DWI, with low signal on ADC map (Solid arrows). **C** depicts an infiltrative mass within the extra- and intra-conal orbit fat on axial T2 (Solid circle). **D** T2 FS coronal images demonstrating loss of flow void and asymmetric enlargement of the left SOV consistent with superior ophthalmic vein thrombosis (Dashed arrow)

**Fig. 4:**
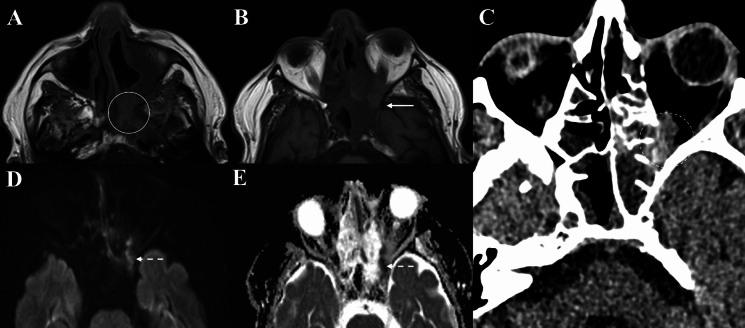
Orbital imaging of a 69-year-old male with invasive aspergillosis of the left orbit. **A** and **B** Demonstrates obliteration of fat planes and an infiltrative mass in the left retro-antral tissues involving the left orbital apex and causing proptosis on axial T1 sequences (Solid circle and arrow). This mass is slightly T1-hyperintense. **C** Depicts bony erosion and hyperdense fungal material within the left ethmoid sinus and orbital apex on axial unenhanced CT (Dashed circle). **D** and **E** Demonstrates regions of diffusion restriction within the left ethmoid sinus and orbital apex corresponding to the area of fungal infection (Dashed arrows)

**Fig. 5:**
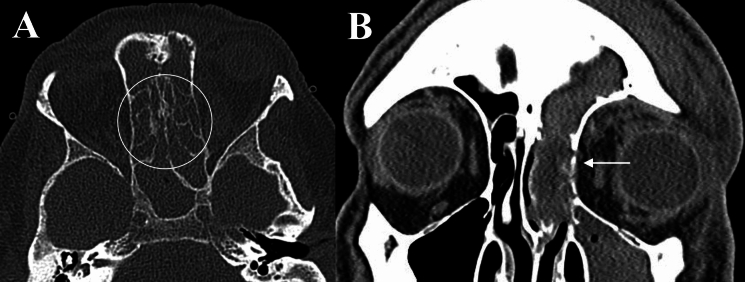
Permeative de-ossification in invasive fungal orbital infections and orbital cellulitis. **A** Demonstrates permeative de-ossification of the ethmoid sinus in axial CT in a 75-year-old male with invasive mucormycosis of the left orbit (Solid circle). **B** Depicts de-ossification of the ethmoid sinus and medial orbital wall in a 28-year-old male with left orbital cellulitis (Solid arrow)

In OC, the ethmoid (11/11, 100%) and maxillary sinuses (10/11, 90.9%), were most commonly affected, with sphenoid sinus (4/11, 36.4%) less commonly involved. Similarly, the ethmoid (11/11, 100%) and maxillary sinus (9/11, 81.8%) were also commonly affected in IFOI and thus, non-differentiating (*p* value = 0.586 and 1.0, respectively). However, the sphenoid sinus (9/11, 81.8%) was more commonly involved in IFOI, albeit not a statistically significant level (*p* value = 0.08). Both entities demonstrated opacification, mucosal-thickening or air-fluid levels indicative of paranasal sinus disease. However, of those with contrast-enhanced imaging, four cases (4/7, 57.1%) of IFOI also demonstrated loss of contrast-enhancement (LoCE) in regions of the paranasal sinus (Figs. 1A, B and 2A). In patients who had DWI sequences, regions of the paranasal sinus in both OC and IFOI demonstrated restricted diffusion, and in some IFOI cases, also extended towards the orbital apex (Figs. 3 and 4). Permeative bony de-ossification (8/11, 63.6%) and bony sclerosis (1/11, 9.1%) were observed in IFOI patients on CT (Figs. 4 and 5). Meanwhile, only 2 IFOI cases (18.2%) demonstrated no orbital bone abnormalities. Eight patients (72.7%) with OC had bony de-ossification, primarily centred along the medial and/or supero- or infero-medial orbital wall.

Pre-septal tissues in OC (11/11, 100%) typically demonstrated contrast-enhancement and heterogenous high T2 signal, but were less commonly involved in IFOI (3/11, 27.3%) (*p* value = 0.001). Meanwhile, proptosis was observed in both OC (8/11, 72.7%) and IFOI (7/11, 63.6%) (*p* value = 1.0). The intra-conal and/or extra-conal orbital fat were involved in both entities. OC demonstrated a predilection to superomedial (4/11, 36.4%) and superolateral (3/11, 27.3%) involvement followed by inferomedial (2/11, 18.2%) and superior (2/11, 18.2%) involvement. Meanwhile, IFOI had primarily medial (4/11, 36.4%) and diffuse (2/11, 18.2%) involvement, followed by superolateral (1/11, 9.1%), inferior (1/11, 9.1%) and inferolateral (1/11, 9.1%) involvement. Isolated orbital apex involvement was involved in two IFOI cases (18.2%). Orbital fat in OC was typically demonstrated by heterogeneous high T2 signal, whilst variable T2 signal (Intermediate or high signal) was observed in IFOI.

**Fig. 6:**
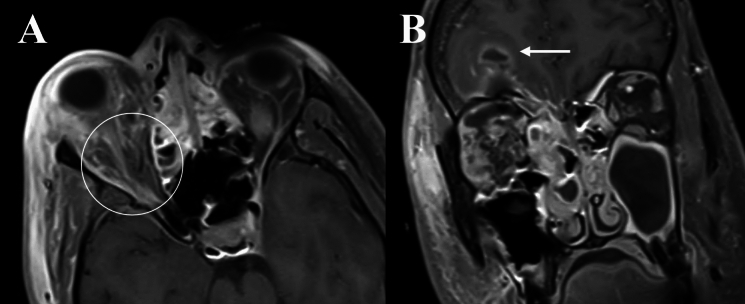
Orbital imaging of a 26-year-old male with right orbital cellulitis. **A** Demonstrates proptosis and, diffuse contrast-enhancement of pre-septal tissues, extra- and intraconal orbit fat on T1 FS CE (Solid circle). There is mucosal thickening in the ethmoid sinuses. **B** Again demonstrates disease in the ethmoidal sinus, along with opacification of the left maxillary sinus. There is a peripherally contrast-enhancing collection in the superior orbit, along with right frontal lobe abscess and sub-galeal abscess on T1 FS CE (Solid arrow)

**Fig. 7:**
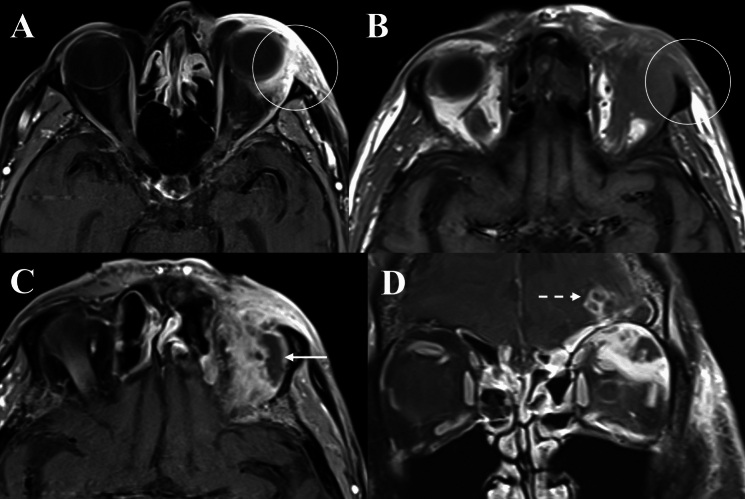
Orbital imaging of a 77-year-old male with invasive aspergillosis involving the left orbit, however, with likely superimposed bacterial orbital cellulitis. **A** and **B** Demonstrate contrast-enhancement and loss of fat plane of the pre-septal tissues and lateral post-septal orbital fat on axial T1 FS CE and T1, respectively (Solid circles). **C** and** D** Demonstrates the lacrimal gland collection (Solid arrow) which is contiguous with intracranial extension and frontal lobe collections (Dashed arrow) on axial and coronal T1 FS CE

The infiltrative lesions in OC were generally localised to the orbit and poorly demarcated, whilst those in IFOI were infiltrative or expansile/mass-like lesions invading the orbital apex from the adjacent paranasal sinuses. Six cases (54.5%) of OC had discrete orbital collection(s) (I.e., subperiosteal abscess or intra-orbital abscess) (Fig. 6). Only one IFOI case (9.1%) demonstrated a lacrimal gland abscess, as evidenced by a peripherally contrast-enhancing collection (Fig. 7) (*p* value = 0.063). This case also demonstrated lacrimal gland enlargement, contrast-enhancement and loss of distinct margins, and was the only case of lacrimal gland involvement observed in IFOI. Meanwhile, lacrimal gland involvement (8/11, 72.7%) was more common in OC, as demonstrated by enlargement, contrast-enhancement and/or loss of distinct margins (*p* = 0.008).

Abnormal EOM involvement in OC was observed as mild diffuse enlargement, contrast-enhancement, loss of distinct margins and heterogenous high T2 signal. EOM involvement in IFOI occurred near the orbital apex as contiguous infiltrative inflammation, typically in the absence of abnormal signal changes, contrast-enhancement or enlargement (due to infiltrative mass obscuring EOM margins). In one IFOI case, there was LoCE of the EOMs at the apex, contiguous with an infiltrative orbital mass from adjacent sinonasal disease (Fig. 1).

Optic nerve and/or sheath changes, as demonstrated by contrast-enhancement on T1 FS CE, were observed in eight (72.7%) cases of IFOI compared to five (45.5%) cases of OC (*p* value = 0.387). Orbital apex involvement in IFOI was typically seen as an invasive infiltrative mass extending from adjacent paranasal sinuses, with/without LoCE. Meanwhile, orbital apex involvement in OC was demonstrated by contrast-enhancement and abnormal T2 signal changes, in the orbital fat and/or EOM.

**Fig. 8:**
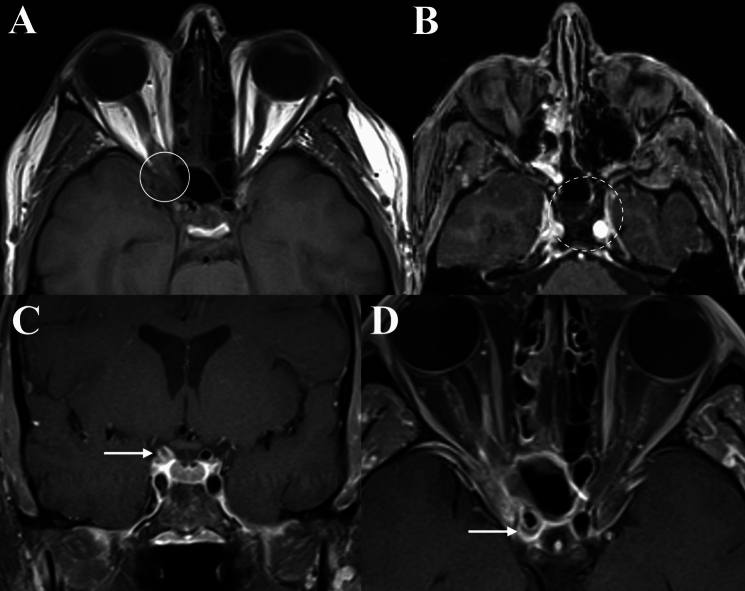
Orbital imaging of a 52-year-old male with invasive aspergillosis affecting the right orbit. **A** Demonstrates orbital apex involvement with adjacent right ethmoid mucosal thickening on axial T1FS (Solid circle). **B** Demonstrates loss of contrast-enhancement of the mucosa of the sphenoid sinus (Dashed circle) on axial T1 FS CE. **C** and **D** Demonstrates vessel wall enhancement of the right internal carotid artery on coronal and axial T1 FS CE (Solid arrows)

Radiological features suggestive of intracranial, and extra-orbital/extra-sinonasal extension in OC were represented by dural enhancement, frontal lobe empyema or collections and observed in nine cases (81.8%) (Fig. 6). Meanwhile, seven cases (63.6%) of IFOI had intracranial extension, contiguous with adjacent paranasal sinus, skull base or involvement of the pterygopalatine fossa, retro-antral and masticator space stranding. Additional radiological abnormalities signifying intracranial extension of invasive fungal infection included cavernous sinus expansion, cavernous sinus thrombosis and vessel wall enhancement (suggestive of vasculitis) (Fig. 3D). In one IFOI case, there was evidence of vasculitis of the right internal carotid artery contiguous with the right cavernous sinus, with the patient subsequently developing a right occipital lobe stroke due to narrowing of the posterior cerebral artery (Fig. 8).

## Discussion

IFOI can have devastating effects if there is delayed diagnosis and initiation of appropriate treatment. Misdiagnosis is common, with incorrect diagnoses ranging from bacterial OC and sinusitis to inflammatory conditions, such as non-specific orbital inflammation [4]. IFOI typically occurs via contiguous spread from the paranasal sinuses and increases mortality compared to orbit-sparing disease [8, 9]. Additionally, the superior orbital fissure and optic canal open directly into the middle cranial fossa and represent avenues of further intracranial extension [9]. There are various mimics of fungal orbital infections and radiological analysis, typically with MRI, remains pivotal in supplementing the clinical assessment.

### Clinical assessment

Firstly, patient demographics and clinical features may assist in differentiating these two entities. Clinical features of invasive fungal rhinosinusitis and bacterial rhinosinusitis with orbital complications have been investigated by Piromchai et al., and findings from our study are largely consistent with prior literature (Table 1) [10–12]. Patients with IFOI were typically older and immunocompromised (eg: haematological malignancy, immunosuppressive medications) or co-morbid with diabetes mellitus. Both OC and IFOI had a male predominance and typically reported orbital pain, periorbital erythema/oedema and proptosis, however, patients with IFOI presented with a more subacute onset of ocular symptoms. Visual compromise, evidenced by reduced visual acuity and/or optic neuropathy, was more common in IFOI compared to OC. Prior studies exploring OC and rhino-orbital-cerebral-mucormycosis (ROCM) on CT suggested that ROCM was more likely to demonstrate EOM limitation, but eyelid swelling was less common [5]. In patients with these risk-factors, naso-endoscopy may help identify an invasive fungal infection, however, the typical grey-black discolouration of nasal mucosa, ulceration and necrotic tissue may occasionally be absent [11, 13]. A higher white-cell count and C-reactive protein was observed in OC, and may be attributable to the high incidence of immunocompromised patients with IFOI, and thus may not reflect disease activity or severity.

### Differentiating radiological features

Typical CT and MRI features of invasive sinonasal fungal infections have been described [13–19]. CT may demonstrate mucosal thickening, either sclerotic or erosive bony changes, fat plane effacement in the pterygoid and infratemporal fossa, and a diffuse contrast-enhancing infiltrative mass invading the orbit. Venous or arterial thrombosis may also occur [2]. Son et al. has previously explored differentiating factors between ROCM and OC on CT, reporting that thickening of the sinus mucosa was more frequent in patients with ROCM compared to OC. However, this study included OC without paranasal sinus disease, whilst our study excluded non-sinogenic OC [5]. MRI may be more sensitive than CT in detecting acute invasive fungal sinusitis and can more readily demonstrate optic nerve and intracranial extension [2, 6, 7].

Within our series, the ethmoid sinus was most commonly involved in both IFOI and OC, consistent with prior studies [5, 12, 20, 21]. In a study of acute invasive fungal rhino-sinusitis, Huang et al. observed higher incidences of frontal, ethmoid and sphenoid sinus involvement in orbital disease, with infection primarily involving the medial and inferior orbit [21]. In IFOI, contrast-enhancement may imply active inflammation, whilst LoCE signifies tissues devitalisation and necrosis, corresponding to higher fungal load with areas of coagulative necrosis [22]. Early MRI features may include oedema in paranasal sinuses and orbital fat, with focal areas of LoCE. LoCE due to fungal invasion and tissue devitalisation, described as the ‘black turbinate sign’, has been noted as an early feature (Fig. 2) [23, 24]. In this study, LoCE in the paranasal sinuses with orbital extension was a specific feature of IFOI, absent in OC. In one case of IFOI, there was LoCE in soft tissues of the right sphenoid sinus and orbital apex contiguous with the pterygopalatine fossa and skull base (Fig. 1). Additionally, LoCE of the EOM at the orbital apex was suggestive of fungal invasion of the EOM, a feature previously described [25].

IFOI and OC had varying patterns of soft-tissue involvement. Firstly, abnormal soft tissue in nasolacrimal ducts, extraconal fat, and pterygopalatine fossa indicates imminent orbital and cerebral invasive fungal infection, and may be demonstrated by loss of normal T1 hyperintensity in the peri-sinus fat planes [17]. Fungal elements may be observed as low to intermediate T1 signal, and are generally T2 hypointense, although intermediate or high signals have occasionally been noted [14–16, 26]. Meanwhile, OC typically demonstrates T2 hyperintensity [12, 26]. Orbital involvement in IFOI was characterised by an infiltrative mass invading the orbital apex, contiguous with affected paranasal sinuses (Table 2). Inflammatory stranding in the pterygopalatine fossa, retro-antral and masticator space were indicative of an invasive fungal infection [17, 18].

**Table 2:** Summary comparing typical radiological features encountered in orbital cellulitis and invasive fungal orbital infections

Radiological feature	Bacterial orbital cellulitis	Fungal orbital infections
Paranasal sinus	Opacification, air-fluid levels, mucosal thickening Bony destruction (CT)	Opacification, air-fluid levels, mucosal thickening LoCE along mucosa Bony destruction (CT)
Pre-septal	Contrast-enhancement and high T2 signal of pre-septal tissues	Typically absent of pre-septal tissue involvement
Orbital fat	Diffuse contrast-enhancement, heterogeneous high T2 signal of orbital fat Prescence of orbital collections	Diffuse infiltrative or expansile/mass-like orbital mass contiguous with adjacent paranasal sinuses Variable T2 signal
Lacrimal gland	Enlargement, contrast-enhancement and/or loss of distinct margins	Uninvolved
EOM	Mild diffuse enlargement, contrast-enhancement, loss of distinct margins and heterogenous high T2 signal	May be difficult to define due to invasive orbital mass LoCE of EOM
Optic nerve/sheath and apex	Contrast-enhancement of optic nerve, sheath and apex	Commonly involved demonstrating contrast-enhancement of optic nerve and sheath Invasive infiltrating mass extending to orbital apex from adjacent paranasal sinuses
Intracranial and extra-orbital/extra-sinonasal extension	Dural enhancement, frontal lobe empyema or collections	Extension to pterygopalatine fossa, retro-antral and masticator space Cavernous sinus expansion, cavernous sinus thrombosis and vessel wall enhancement

*CT*: Computed tomography, T2- T2-weighted imaging, *LoCE*: Loss of contrast-enhancement, *EOM*: extraocular muscles

A range of radiological features were observed depicting intracranial and/or extra-orbital/extra-sinonasal extension in OC and IFOI (Table 2). Intracranial extension in IFOI was represented by dural thickening and cavernous sinus expansion. Meanwhile, in OC there was dural thickening and/or contrast-enhancement, cerebritis and frontal lobe abscesses (demonstrating diffusion restriction on DWI). These findings are typical for each disease. The authors draw attention to the case depicted in Fig. 7, whereby MRI demonstrated abnormalities suggestive of an IFOI with superimposed bacterial OC. Firstly, LoCE in the left ethmoid region and permeative bony de-ossification of the frontal sinus, along with microbiological confirmation of intraoperative specimens, were consistent with an invasive orbital aspergillosis. However, this case also demonstrated superolateral orbital fat cellulitis, a lacrimal gland abscess (uncommon in IFOI) and a left frontal lobe abscess, as indicated by a peripherally contrast-enhancing lesion (Fig. 7). In its early stages, bacterial abscesses are seen as T1 hypointense and T2 hyperintense areas with minimal or heterogeneous contrast-enhancement [19]. Typically, fungal abscesses demonstrate a hypointense core with a surrounding iso- to mildly hyper-intense rim, with peripheral contrast-enhancement [2]. Almost all fungal abscesses show non-enhancing intra-cavitory projections directed centrally from the wall, and there may be high T2 signal in the core with a surrounding hypointense rim [27]. These features were not observed in the intra-orbital collection. The outer margin of the pyogenic abscess wall will be smooth or lobulated, in contrast to fungal lesions which will have crenated wall in more than half of the abscesses [19, 27]. A distinct feature which may differentiate between bacterial from fungal abscesses is the ‘dual rim’ sign on DWI, however DWI was not performed in this case [28]. Furthermore, pyogenic abscesses show diffusion restriction whereas fungal lesions may have variable diffusibility (i.e. variable DWI and ADC) [29]. These differences in radiological appearance may be attributed to the different composition of fungal abscesses. Fungal abscesses are typically multiple and can involve the basal ganglia, whilst bacterial abscesses are often solitary lesions sparing the basal ganglia [19, 30].

The expedient diagnosis of IFOI can be a complex challenge, due to variable patient risk factor, co-morbidities, clinical course and response to initial treatment, guiding further investigation and commencement of empirical treatment. Ultimately, the presence of the aforementioned radiological features are indicative of an invasive fungal infection and may help to differentiate IFOI from sinogenic OC. The presence of these extensive and/or specific radiological features in high-risk patient demographics (e.g.: highly immunocompromised, patients with poorly controlled diabetes mellitus) should prompt endoscopic evaluation and/or early surgical management to obtain intraoperative microbiological confirmation of an invasive fungal pathology [25, 31–34]. The most frequently affected sites, and hence the most sensitive for biopsy are the middle turbinate, nasal septum and floor of the nasal cavity [1, 13, 35].

### Prognosis

Patients with IFOI demonstrated significantly worse prognosis, including visual compromise and mortality. In many cases, IFOI led to devastating and life-threatening outcomes, with many patients proceeding to enucleation. Meanwhile, prognosis in OC was more favourable, with majority of patients achieving complete resolution following an appropriate course of IV/oral antibiotics. The utility of pre-intervention MRI features in predicting visual and mortality outcomes in acute invasive fungal rhinosinusitis has been investigated [20]. In Idowu et al.’s study, orbital apex and cerebral artery (i.e. arteritis/arterial occlusion) involvement were risk factors for poor visual outcomes. Visual compromise in IFOI may be due to a combination of direct optic nerve invasion, ischemic and/or compressive optic neuropathy [36, 37]. Meanwhile, increased mortality was associated with facial soft tissue and nasolacrimal apparatus involvement.

There are several study limitations pertaining to the retrospective nature and small sample size, with limitations on statistical power. Additionally, at our tertiary institution, MRI scans were typically reserved for OC whereby there was diagnostic uncertainty and/or suspicion of severe complications, such as suspected intracranial extension. This may be reflected in the higher incidence of superior orbital collections and involvement of the superior muscle complex, supero-medial and supero-lateral orbital fat involvement in cases of OC. Thus, the inclusion criteria may depict cases of greater disease severity [38]. However, the authors note that cases of severe sinogenic OC encountered are more likely to reflect realistic clinical scenarios whereby expedient differentiation from IFOI is necessary and more challenging.

## Conclusion

In conclusion, this study provides a descriptive characterisation of the radiology of IFOI on MRI and summarises key orbital and extra-orbital features that may differentiate this entity from OC. This study identifies the specific constellation of radiological abnormalities, such as LoCE of the paranasal and orbital soft tissues, the location and pattern of contiguous soft-tissue involvement, that may help provide expedient identification of IFOI, which ultimately necessitate early surgical intervention to obtain a microbiological specimen.

## Data Availability

The data that support the findings of this study are available from the corresponding author, [TA], upon reasonable request.
